# Expression and Functional Characterization of Various Chaperon-Usher Fimbriae, Curli Fimbriae, and Type 4 Pili of Enterohemorrhagic *Escherichia coli* O157:H7 Sakai

**DOI:** 10.3389/fmicb.2020.00378

**Published:** 2020-03-20

**Authors:** Laura Elpers, Michael Hensel

**Affiliations:** ^1^Abteilung Mikrobiologie, Osnabrück University, Osnabrück, Germany; ^2^CellNanOs – Center of Cellular Nanoanalytics Osnabrück, Osnabrück University, Osnabrück, Germany

**Keywords:** EHEC O157:H7, Sakai, fimbriae, Curli, type 4 pili

## Abstract

Enterohemorrhagic *Escherichia coli* (EHEC) is a highly pathogenic strain leading to hemorrhagic colitis and to the hemolytic-uremic syndrome (HUS) in humans. The mechanisms by which pathogenic *E. coli* infect and colonize humans leading to the typical disease pattern are in focus of many investigations. The adhesion of EHEC to epithelial cells by the coordinated translocation of receptor Tir and surface expression of corresponding adhesin intimin is a key event in host–pathogen-interaction. However, less is known about other adhesins encoded by EHEC, especially about the complex set of fimbrial adhesins varying among various serotypes. Here, we investigate EHEC serotype O157:H7 strain Sakai possessing at least 16 putative fimbrial gene clusters. Using a synthetic heterologous expression system in a non-pathogenic *E. coli* strain, a subset of 6 gene clusters for fimbrial adhesins was analyzed. We were able to visualize surface expression of two γ1 class fimbriae (Fim and Ycb), two γ4 class fimbriae (Yad and Yeh), and two fimbrial adhesins which are assembled by the nucleation/precipitation pathway (Curli fimbriae), and by a type 2 secretion system (type 4 pili). Further, we elucidated the impact of these fimbrial adhesins in adhesion to various epithelial cells lines (HeLa, MDCK, and CaCo2), and the contribution on biofilm formation. We demonstrate the ultrastructure of Fim fimbriae and Yad fimbriae of EHEC Sakai, and Yeh fimbriae of *E. coli* in general. The involvement of Fim fimbriae of EHEC Sakai to adhesion to various epithelial cell lines, and contribution to biofilm formation is reported here. Our approach provides first ultrastructural and functional data for novel EHEC adhesins, and enables further understanding of the involvement of fimbrial adhesins in pathogenesis of EHEC Sakai.

## Introduction

Over the last 30 years, enterohemorrhagic *Escherichia coli* (EHEC) serotype O157:H7 Sakai was the causing agent of hundreds of outbreaks of hemorrhagic colitis and hemolytic-uremic syndrome (HUS). The largest outbreak occurred in 1996 in Sakai City, Japan with over 6,000 reported cases of infection. Approximately 1,000 patients were hospitalized, including 100 patients with HUS leading to three deaths (Hayashi et al., 2001). More recent outbreaks with smaller numbers of patients occurred in 2015 in England, where *E. coli* O157:H7 Sakai was associated with salad leaves in a mixed leaf prepacked salad (Mikhail et al., 2018), and in 2016 in Kanagawa, Japan, where minced meat cutlets were contaminated with *E. coli* O157:H7 Sakai (Furukawa et al., 2018).

The pathogenicity of *E. coli* O157:H7 Sakai relies on various virulence factors. The genetic locus of enterocyte effacement (LEE) harbors various virulence genes known to lead to attaching and effacing (A/E) lesions. These A/E lesions are caused by a type 3 secretion system (T3SS) which translocates the intimin receptor Tir, and effector proteins to host cells. Subsequently, the adhesin intimin (*eae*) binds to Tir, resulting in the tight attachment and formation of pedestals. EHEC strains typically cause HUS by Shiga toxin Stx, which leads to the inhibition of protein synthesis and cell death, especially in the renal tubular cells and microvascular endothelial cells (Clements et al., 2012). Further, the *E. coli* O157:H7 Sakai-specific plasmid pO157 encodes several virulence factors including hemolysin *ehxA*, putative adhesin *toxB* and serine protease *espP*. However, *E. coli* O157:H7 Sakai possesses a complex set of adhesive structures including various fimbrial operons. This set comprises 16 different fimbrial gene clusters: *yeh*-like, *yad*, *sfm*, *ybg*, *ycb*, type 3-like, Curli, F9, *yeh*, *yfc*, *yra*, *lpf*-like (*lpf1*), *lpf* (*lpf2*), *fim*, *mat* fimbriae and type 4 pili (Low et al., 2006). Twelve of these gene clusters were predicted to encode chaperon-usher fimbriae. In comparison to non-pathogenic *E. coli* strain K-12, harboring 12 fimbrial operons, some fimbrial gene clusters are also found in pathogenic strain *E. coli* O157:H7 Sakai (Korea et al., 2010; Wurpel et al., 2013). Low et al. (2006) demonstrated by *lacZ* promoter fusions the expression of F9 and Yeh fimbriae after 24 h static growth in LB medium at 37°C and enhanced expression by a lower temperature of 28°C. Yet for most of these fimbrial adhesins, little is known about native expression conditions and thus knowledge of their role in virulence for EHEC Sakai is sparse.

The most prominent fimbrial adhesins so far are both types of Lpf fimbriae. This adhesins are expressed at late log phase in cell culture media at 37°C, pH 6.5 (McWilliams and Torres, 2014), bind laminin and fibronectin (Farfan et al., 2011), and are involved in colonization of the intestine of rabbits (Lloyd et al., 2012a). Curli fimbriae were investigated in several studies that revealed the involvement of Curli fimbriae in adhesion to abiotic surfaces, i.e., polystyrene, glass and rubber (Pawar et al., 2005) and biotic surfaces, i.e., alfalfa sprouts (Lloyd et al., 2012b). Furthermore, expression of Curli fimbria was shown during interaction with lettuce leaves (Fink et al., 2012), and after static growth in LB medium for 24 h at 37°C.

However, less is known about the other fimbriae encoded by *E. coli* O157:H7 Sakai. Therefore, it is of crucial importance to investigate these adhesive structures to gain insights into further hosts that may form sites of contamination, and routes of infection. We decided to clone selected fimbrial adhesins for expression under control of the Tet-on system to achieve regulated expression under laboratory conditions. In this study, two γ1 fimbriae (i.e., Fim and Ycb), two γ4 fimbriae (i.e., Yad and Yeh), Curli fimbriae assembled by the nucleation/precipitation pathway, and type 4 pili assembled by type 2 secretion system were surface-expressed and visualized. The contribution of these fimbriae to biofilm formation and autoaggregation was determined. Using a luciferase-based adhesion assay, we investigated the role of the adhesins in binding to various types of mammalian host cells including HeLa, MDCK, and Caco2 cells. We investigated non-polarized HeLa cells (human cervix epithelial cells) as standard cell culture model for host–pathogen interaction. MDCK cells (Madin-Darby Canine kidney cells) are routinely used for polarized epithelial cell models. Caco2 cells are polarized epithelial cells from human colonic cancer, and represent the native infection site of *E. coli* O157:H7 Sakai in the human host.

## Results

Since for most adhesins of *E. coli* O157:H7 Sakai the conditions for native expression are not known, we decided to express the genes of interest ectopically under control of a tetracycline-inducible promoter, as described in Hansmeier et al. (2017). This Tet-on approach enables the analysis of various fimbrial operons, Curli fimbriae and type 4 pili of *E. coli* O157:H7 Sakai in the non-pathogenic *E. coli* strain ORN172 (Woodall et al., 1993). Strain ORN172 is deleted in Fim (also referred to as type I) fimbriae (*fimBEACDFGH*), and under the experimental conditions used here, was devoid of adhesive structures on the bacterial surface.

### Ultrastructural Analyses of *E. coli* O157:H7 Sakai Fimbriae

**FIGURE 1:**
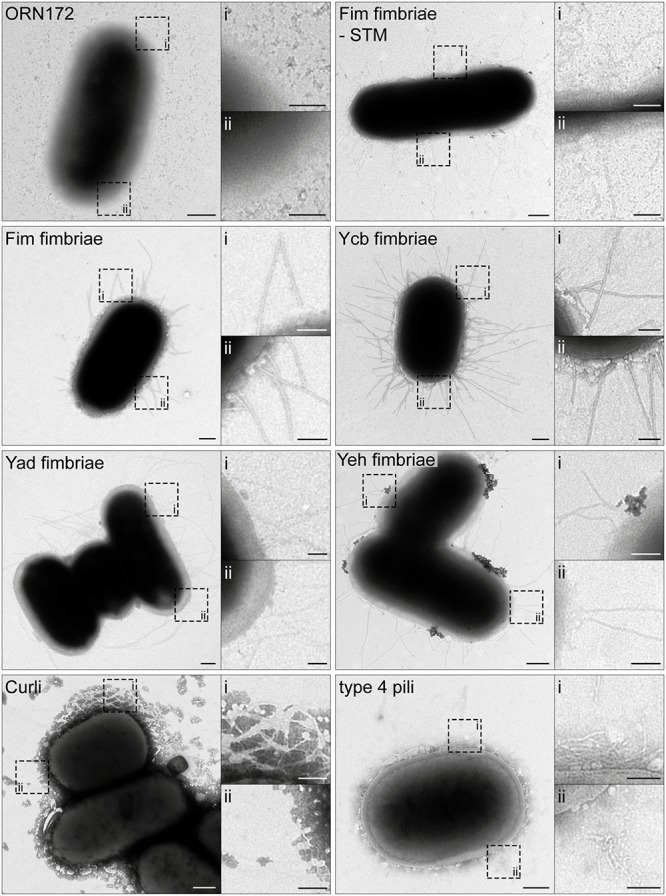
Ultrastructure of *Escherichia coli* O157:H7 Sakai fimbriae after surface expression by *E. coli* ORN172. *E. coli* ORN172 was used as host strain for expression of Fim-STM as positive control, and various *E. coli* O157:H7 Sakai fimbriae as indicated. ORN172 indicates the negative control with vector only. Each panel contains an overview of bacteria, and two hatched boxes indicate enlarged details (i and ii). Only AHT-induced strains are displayed, and non-induced negative controls are shown in Supplementary Figure S1. Scale bars, 250 and 100 nm in overviews and details, respectively.

Since for many adhesins of *E. coli* O157:H7 Sakai detection by specific antibodies is not available, we analyzed surface expression of various adhesins by transmission electron microscopy (TEM). Bacterial strains were subcultured for 3.5 h with or without 10 ng/ml anhydrotetracycline (AHT) to induce expression of adhesins, and further processed for microscopy. As control, heterologous expression of Fim fimbriae of *Salmonella enterica* serovar Typhimurium (STM) in ORN172 was used. Visualization of C/U-assembled Fim fimbriae and Ycb fimbriae of *E. coli* O157:H7 Sakai revealed rigid fimbrial structures distributed over the whole bacterial surface (Figure 1). The length of fimbrial filaments varied, likely due to different phases of fimbriae assembly. Compared to Fim and Ycb fimbriae, Yad (sometimes termed Ecp fimbriae, due to incorect genome annotation of Yad fimbriae usher as *ecpD*) and Yeh fimbriae revealed thinner and more flexible structures. In contrast to the rigid Fim and Ycb fimbriae, Yad and Yeh fimbriae often showed filaments that stick together or crossed, resulting in a branched appearance.

Curli fimbriae, assembled by the nucleation/precipitation pathway, exhibited a completely different morphology. Here we observed the branched distribution of Curli fimbriae over the bacterial surface, thereby possibly trapping extracellular material. Expression of type 4 pili (also called type IV pili or Hcp – hemorrhagic coli pilus) led to an unstructured meshwork distributed over the bacterial surface. We could not observe clustering of type 4 pili leading to distinct long bundles of type 4 pili. For all constructs, no adhesive structures were observed on the bacterial surface in absence of inducer AHT (Supplementary Figure S1).

### Impact of *E. coli* O157:H7 Sakai Fimbriae in Adhesion to HeLa, MDCK, and Caco2 Cells

For high-throughput analysis of the contribution of *E. coli* O157:H7 Sakai fimbriae to adhesion to various cell lines, we established an adhesion assay based on quantification of adherent bacteria by bioluminescence. To generate a bioluminescent host strain, we used the low-copy number plasmid pGEN-*lux* encoding *luxCDABE* under control of constitutive promoter P_EM__7_ (Lane et al., 2007). Expression of luciferases LuxA and LuxB, as well as enzymes LuxC, LuxD, and LuxE, resulted in constitutively bioluminescent bacteria. The level of relative bioluminescence was dependent on the amounts of bacteria (Supplementary Figure S2A). For our analyses, we exchanged the carbenicillin resistance of pGEN-*lux* by chloramphenicol resistance (amplified from pKD3) to enable plasmid maintenance with plasmids for Tet-on expression of *E. coli* O157:H7 Sakai fimbriae in ORN172. The newly constructed plasmid p5236 or short [Lux] harbored P_EM__7_::*luxCDABE* P_*cat*_::*cat* and exhibited in ORN172 levels of bioluminescence comparable to pGEN-*lux* in TOP10 (Supplementary Figure S2A). To test proper AHT-induced expression of fimbriae in presence of [Lux], we checked synthesis and assembly of Fim fimbriae of STM (Fim-STM) in ORN172 [Lux] by flow cytometry. In AHT-induced cultures, synthesis and surface expression of Fim fimbriae were observed, whereas in background strain ORN172 [Lux], and in the non-induced cultures, no Fim fimbriae were detected (Supplementary Figure S3).

Next, HeLa, MDCK, or Caco2 cells were grown in 96-well plates, infected with strains with or without expression of various *E. coli* O157:H7 Sakai fimbriae, and bioluminescence of adhering bacteria were measured in microplate reader. We tested if results of luminescence (Lux)-based adhesion assays were comparable to results for CFU-based adhesion assays with MDCK cells (Supplementary Figures S2B,C). The assay (Supplementary Figure S2B) resulted in the typical phenotype of adhesion to epithelial cells mediated by Fim fimbriae of STM (Yue et al., 2015). We detected higher adhesion rates in Lux-based adhesion assay compared to CFU assays, which was adjusted by normalization of ORN172 [Lux] to 100% (Supplementary Figure S2C). The number of washing steps with PBS required to remove non-adherent bacteria was controlled in Supplementary Figure S2D. We determined that three washing steps were sufficient to obtain a clear distinction between Fim fimbriae expressing and non-expressing bacteria in adhesion to MDCK cells.

Furthermore, we tested the ability of ORN172 [Lux] to adhere to, and to invade HeLa and Caco2 cells in assays at various temperatures (4°C, RT, 37°C; Supplementary Figures S2E,F). STM WT and STM Δ*invC* (defective in invasion) were used as controls. For ORN172 [Lux] strains no invasion was observed for either cell line, whereas STM WT was able to invade HeLa and Caco2 cells. Comparison of adhesion rates at various temperatures showed highest binding to HeLa and Caco2 cells mediated by Fim fimbriae of STM at RT or 37°C. Due to the standard growth temperature for the various cell lines we decided to perform the Lux-based adhesion assays at 37°C.

**FIGURE 2:**
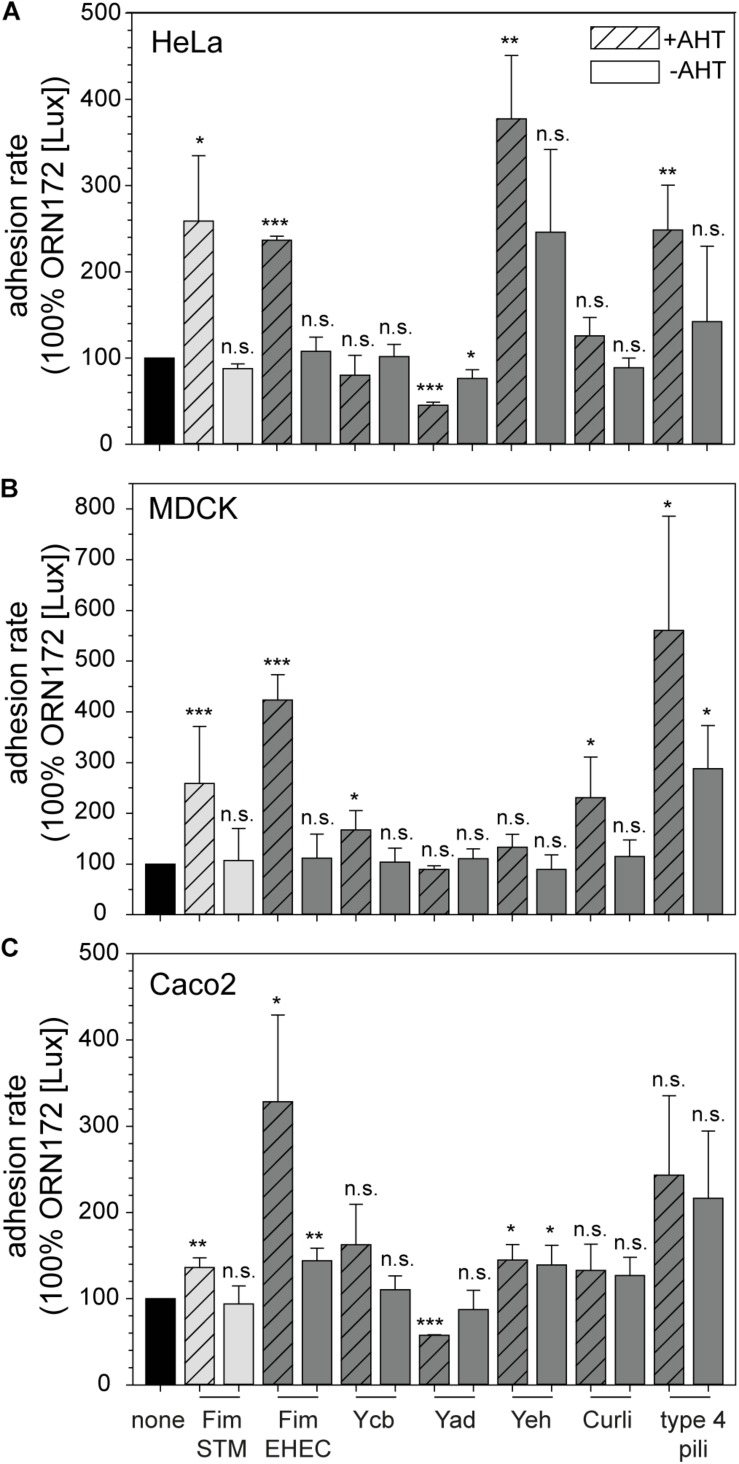
Contribution of *E. coli* O157:H7 Sakai fimbriae to adhesion to HeLa, MDCK, and Caco2 cells. HeLa **(A)**, MDCK **(B)**, or Caco2 cells **(C)** were infected with *E. coli* ORN172 [Lux] constitutively expressing luciferase and harboring the vector (none, black bars), or plasmids for expression of STM Fim fimbriae (Fim STM, light gray bars), or various fimbriae of *E. coli* O157:H7 Sakai (dark gray bars). If indicated by +AHT, expression of fimbriae was induced by addition of 10 ng/ml AHT and subculture for 3.5 h. Non-induced controls (–AHT) were incubated in parallel. Host cells were infected with various strains at a MOI of 25, and infection was synchronized by centrifugation for 5 min at 500 × *g*. After infection for 55 min at 37°C, cells were washed three times with pre-warmed PBS or medium, and luminescence was measured using a microplate reader. Adhesion rates were calculated by the quotient of initial relative luminescence, and luminescence after infection and washing normalized to 100% ORN172 [Lux] without expression of fimbriae. Shown are means and standard deviations of at least three biological replicates. Statistical significances were calculated by Student’s *t*-test and are indicated as follows: n.s., not significant; *p < 0.05, **p < 0.01, ***p < 0.001.

Fim fimbriae of STM contribute to adhesion to various cells lines, e.g., HeLa, MDCK, and Caco2, and were therefore used as control in all Lux-based adhesion assays. Expression of Fim fimbriae of *E. coli* O157:H7 Sakai in ORN172 [Lux] led to significant increased adhesion level to all three cell lines compared to background strain ORN172 [Lux] (Figure 2; means of 237% for HeLa, 423% for MDCK, and 328% for Caco2). The non-induced cultures of ORN172 [Lux] [Fim] showed no increased adhesion to HeLa and MDCK cells, but revealed a significant higher adhesion level to Caco2 cells compared to background strain (Figure 2C, mean 144%). However, the increased adhesion by AHT-induced expression of Fim fimbriae in ORN172 [Lux] to Caco2 cells is significant higher compared to non-induced bacteria (*p* = 0.035). Moreover, surface expression of Fim fimbriae was observed by TEM only after AHT induction (Figure 1). The other γ1-class fimbriae analyzed in this study, Ycb fimbriae, revealed a significantly increased adhesion to MDCK cells (mean 167%), and a non-significantly increased adhesion to Caco2 cells (mean 162%). Again, non-induced cultures did not alter adhesion levels. Increased adhesion to MDCK and Caco2 cells by expression of Ycb fimbriae suggest that Ycb fimbriae are specifically involved in adhesion to polarized epithelial cells. Expression of Yad fimbriae did not alter adhesion to MDCK cells, but exhibited significant decreased adhesion levels to HeLa and Caco2 cells (means of 45 and 57%, respectively). Hence Yad fimbriae may interfere with other features of ORN172 [Lux] leading to basal adhesion to HeLa and Caco2 cells. Expression of Yeh fimbriae, belonging to γ4 class of fimbriae like Yad fimbriae, did not alter adhesion to MDCK cells. Although expression of Yeh fimbriae led to significant increased adhesion to Caco2 cells in comparison to ORN172 [Lux] (mean 145%), adhesion levels were not significantly increased in comparison to non-induced bacteria (mean 139%). However, expression of Yeh fimbriae was only observed after AHT-induction (Figure 1). For Yeh fimbriae expression, increased adhesion to HeLa cells was revealed (mean 377%). Expression of Curli fimbriae, which are assembled by the nucleation/precipitation pathway, only led to an increased adhesion to MDCK cells (231% mean). Adhesion to HeLa and Caco2 cells was not affected by expression of Curli fimbriae. AHT-induced expression of type 4 pili resulted in increased adhesion levels to HeLa and MDCK cells (means of 248 and 560%, respectively). Infection of MDCK cells with non-induced bacteria to MDCK cells also resulted in a significant increased adhesion (mean 288%), even though surface expression of type 4 pili was dependent of AHT induction (Figure 1).

### Involvement of *E. coli* O157:H7 Sakai Fimbriae in Biofilm Formation

**FIGURE 3:**
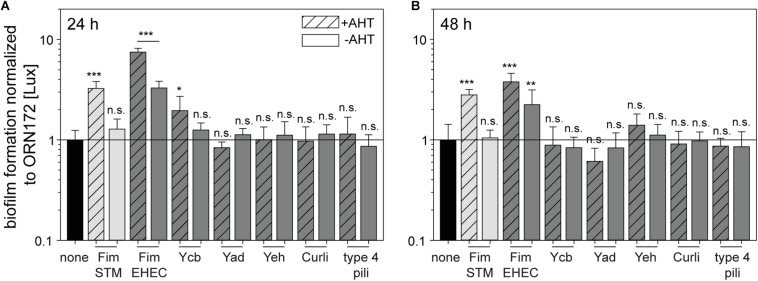
Contribution of *E. coli* O157:H7 Sakai fimbriae to biofilm formation. Expression of fimbriae were induced by 10 ng/ml AHT for 3.5 h in subculture. Bacteria were diluted to 3 × 10^8^ bacteria/ml in LB with chloramphenicol, or LB with chloramphenicol and carbenicillin. Bacteria were incubated in 96-well plates in technical triplicates for 24 h **(A)** or 48 h **(B)** at 37°C in a humidified chamber. As control, wells with media without inoculum were used. After incubation, wells were washed and biofilm components were stained by crystal violet. Absorbance at 595 nm was determined using a multi-well plate reader. Shown are means of at least three biological replicates with technical triplicates represented as bar with standard deviation. Rates of biofilm formation of *E. coli* ORN172 [Lux] expressing various fimbriae of *E. coli* O157:H7 Sakai (dark gray bars), STM Fim fimbriae (light gray bars), or no fimbriae (none, black bars) were analyzed for statistical significances as described in Figure 2.

An important factor of bacterial pathogenesis is formation of biofilms. Thereby, bacteria are able to evade unfavorable environmental conditions (e.g., host defense, nutrient deficiency, and antibiotics) and to persist on biotic and abiotic surfaces. The biofilm consists of a community of bacteria within an extracellular matrix composed of exopolysaccharides, proteins and nucleic acids. The first step in biofilm formation is the attachment to biotic or abiotic surfaces, possibly mediated by fimbriae or other adhesive structures on the bacterial surface. Further, these adhesive structures can be used to build part of the extracellular matrix (Zogaj et al., 2001). The contribution of *E. coli* O157:H7 Sakai fimbriae in biofilm formation on non-treated polystyrol was investigated by a crystal violet assay in this study. Therefore, cultures with or without AHT induction were diluted in LB and incubated in 96-well plates at 37°C for 24 h or 48 h. Non-adherent bacteria were removed by washing and biofilms were stained with crystal violet. Subsequently, OD_595_ was measured and relative biofilm formation was quantified and normalized to background strain ORN172 [Lux]. For control, ORN172 [Lux] expressing Fim fimbriae of STM was used while contributing biofilm formation. Increased biofilm formation was observed after 24 and 48 h, whereas non-induced cultures did not alter relative biofilm formation compared to ORN172 [Lux] (Figures 3A,B). Expression of Fim fimbriae of *E. coli* O157:H7 Sakai led to an increased biofilm formation after 24 h and 48 h incubation (7.5- and 3.8-fold, respectively). Non-induced bacteria as well showed an increased biofilm formation. However, in both cases expression of Fim fimbriae induced by AHT still led to a significant higher biofilm formation compared to non-induced cultures (24 h *p* = 0.0004; 48 h *p* = 0.029). The expression of Ycb fimbriae led to increased biofilm formation after 24 h (twofold), while no further impact was observed for 48 h incubation. Expression of Yad fimbriae, Yeh fimbriae, Curli fimbriae and type 4 pili did not alter biofilm formation compared to background strain. Biofilms formed by *E. coli* strains contain biofilm matrix components such as cellulose or colanic acid, in addition to proteinaceous matrix components such as Fim or Curli fimbriae. The expression of these additional matrix components is under control of the master regulator CsgD, and increased at 30°C compared to 37°C (Zogaj et al., 2001; Gerstel and Römling, 2003). We analyzed contribution of Tet-on expression of *E. coli* O157:H7 Sakai Curli to biofilm formation at 30°C. We observed that expression of Fim fimbriae of STM increased biofilm formation about 2-fold after 24 h incubation, and a stronger increase (ca. 3.2-fold) was determined for assays with *E. coli* O157:H7 Sakai Curli (Supplementary Figure S4). Biofilm assays performed for 72 h did only reveal minor effects, probably due to the loss of AHT induction. The data indicate that Tet-on expression of *E. coli* O157:H7 Sakai Curli results in biofilm formation if other matrix components are provided by conditions of native expression.

### Expression of Curli Fimbriae Leads to Bacterial Autoaggregation

Adhesion to various cell lines and the attachment to biotic and abiotic surfaces can be affected by bacterial autoaggregation. Autoaggregation can be caused by homo- or heterotypic interactions of bacterial surface structures such as fimbriae. Therefore, we investigated potential autoaggregation evoked by the expression of *E. coli* O157:H7 Sakai fimbriae, Curli fimbriae and type 4 pili. Light microscopy analysis was performed with subcultures used for Lux-based adhesion assay diluted to ca. 3 × 10^8^ bacteria/ml in PBS. Autoaggregation was observed solely for Curli-expressing bacteria (Supplementary Figure S5). No bacterial autoaggregation was detectable for ORN172 expressing Fim fimbriae, Ycb fimbriae, Yad fimbriae, Yeh fimbriae, or type 4 pili.

## Discussion

The highly variable and complex sets of fimbrial adhesins present in non-pathogenic and pathogenic *E. coli* strains are only poorly understood. Especially for pathogenic strains, such as *E. coli* O157:H7 Sakai causing life-threatening infections, it is crucial to unravel the functions of all potential virulence factors to gain a better understanding of pathogenesis. In this study, we investigated six operons encoding putative fimbrial adhesins of *E. coli* O157:H7 Sakai. Surface expression of all investigated adhesins was demonstrated by ultrastructural analyses. Both γ1 fimbriae, i.e., Fim and Ycb, showed rigid fimbriae typical for γ1 class fimbriae (Nuccio and Bäumler, 2007). Fim fimbriae of *E. coli* O157:H7 Sakai were visualized here for the first time, and reveal a similar ultrastructure as observed for *E. coli* K-12 Fim fimbriae (Korea et al., 2010). In this study, Ycb fimbriae appeared to possess a rigid structure, in line with characteristics of γ1 class fimbriae. However, Samadder et al. (2009) observed a more flexible structure of Ycb fimbriae. Yad and Yeh fimbriae revealed a flexible structure which is characteristic for γ4 class fimbriae (Nuccio and Bäumler, 2007). Here we visualized, to our knowledge for the first time, surface-expressed Yeh fimbriae of *E. coli*. The Yad fimbriae of *E. coli* O157:H7 Sakai showed a similar appearance compared to Yad fimbriae encoded by *E. coli* K-12 (Korea et al., 2010; Larsonneur et al., 2016). Expression of Curli fimbriae and type 4 pili revealed diffuse adhesive structures on the bacterial cell surface. Curli fimbriae, assembled by the nucleation/precipitation pathway, are known to build a meshwork of Curli fimbriae (Kay et al., 2017), whereas type 4 pili (Xicohtencatl-Cortes et al., 2009) can assemble and form pili bundles. While heterologous Tet-on expression of *E. coli* O157:H7 Sakai Curli fimbriae resulted in a comparable meshwork, surface-expressed *E. coli* O157:H7 Sakai type 4 pili did not show bundling. This difference may be due to lower levels of expression in the heterologous system, and/or lack of additional factors that are co-expressed under native conditions.

The functional expression of these fimbrial structures of *E. coli* O157:H7 Sakai enabled us to demonstrate the involvement of fimbriae in adhesion to various epithelial cell types (HeLa, Caco2, and MDCK), and further involvement in biofilm formation. Fim fimbriae had impact in adhesion to all three cell lines and in biofilm formation, which is independent from autoaggregation. Expression of Fim fimbriae encoded by *E. coli* O157:H7 Sakai was not observed under laboratory conditions. Due to the loss of the 16 bp phase switch in front of the promoter of *fimA*, expression regulation remains in off state (Iida et al., 2001; Cookson et al., 2002). Thus, Fim fimbriae are often neglected in studies of *E. coli* O157:H7 Sakai fimbriae (Xicohtencatl-Cortes et al., 2007). However, some *E. coli* O157:H7 Sakai isolates showed expression of Fim fimbriae after serial passages. If expressed, Fim fimbriae were shown to mediate adhesion to rat intestinal mucins, probably by binding the link glycopeptide, being the only mucin component known to harbor mannose (Sajjan and Forstner, 1990). This study reveals the importance of studying all fimbrial operons with special attention to variations between single isolates of *E. coli* O157:H7 Sakai. This is also supported by the fact that a single amino acid exchange from N to K in FimH occurred, leading to less binding to uroepithelial cells (Shaikh et al., 2007). In contrast, Fim fimbriae of uropathogenic *E. coli* (UPEC) strains are known to be a major virulence factor for adhesion to uroepithelial cells. Therefore, the amino acid exchange probably enables infection of the intestine, further revealed by the binding properties of Fim fimbriae to HeLa, Caco2, and MDCK cells in this study.

The further γ1 class fimbriae investigated in this study are Ycb fimbriae. We demonstrate the involvement of Ycb fimbriae in the early phase of biofilm formation, i.e., after 24 h incubation under our experimental conditions. Increased biofilm formation was also observed by Korea et al. (2010) for Ycb fimbriae encoded by *E. coli* K-12, who also demonstrated surface expression by TEM, and observed similar rigid structures as shown here for Ycb fimbriae of *E. coli* O157:H7 Sakai. Ycb fimbriae were found to bind to several cell types including human HEp-2 and HT-29 and further MDBK cells (Samadder et al., 2009), whereas expression of *E. coli* K-12 Ycb fimbriae revealed decreased adhesion to bladder cells T24 (Korea et al., 2010). We only detected significant involvement of Ycb fimbriae of *E. coli* O157:H7 Sakai in adhesion to MDCK cells, and a slight increase in adhesion to Caco2 cells, probably highlighting the importance of Ycb fimbriae in intestinal infection. A *ycb* mutant strain was not altered in binding to spinach leaves (Saldana et al., 2011), and the binding properties of Ycb fimbriae to laminin (Samadder et al., 2009) and heparin (Hsiao et al., 2016) further support a role in intestinal infection.

Two γ4 class fimbriae were investigated in this study. Genes for *yad* fimbriae are present in several serotypes of *E. coli*, ranging from non-pathogenic *E. coli* K-12 to pathogenic serotypes UPEC, APEC, and EHEC (Wurpel et al., 2013). Expression of Yad fimbriae of *E. coli* K-12 was observed at ambient temperatures (RT), under anaerobic conditions, as well as during biofilm formation (Larsonneur et al., 2016). In contrast, Yad fimbriae encoded by APEC strain SCI-07 causing chicken infections, are expressed at higher temperatures of 41°C (Verma et al., 2016). In *E. coli* O157:H7 Sakai, expression of *yad* fimbriae is induced by acid stress comparable to conditions during the passage through the stomach in the human host. The varying conditions for expression of *yad* fimbriae of *E. coli* strains are probably indicative for adaptations to their specific hosts, it is comprehensible that the impact of Yad fimbriae in adhesion to different cells varies. For Yad fimbriae synthesized by *E. coli* O157:H7 Sakai it was shown that adhesion to Caco2 cells was enhanced only under acid stress. Expression of Yad fimbriae under non-stressed conditions showed no enhanced adhesion to Caco2 cells (Chingcuanco et al., 2012). This is partly in line with our observation that surfaces expression of *Yad* fimbriae decreased adhesion to Caco2 and HeLa cells. However, data on binding specificities of Yad fimbriae are still elusive for any serotype.

Yeh fimbriae are further members of the γ4 class fimbriae which were visualized here for the first time. We revealed a contribution of Yeh fimbriae in adhesion to HeLa cells, while adhesion to polarized cells was not affected. Expression analyses by β-galactosidase assays revealed an expression of *yeh* genes in LB in stationary culture as well as in static cultures, while expression was increased at lower temperatures. In addition, expression was observed during biofilm formation at 37°C and 28°C (Low et al., 2006). We did not observe contribution to biofilm formation by expression of *E. coli* O157:H7 Sakai *yeh* fimbriae. We conclude that expression of *yeh* under static conditions (i.e., during biofilm formation at 37°C) and lower temperatures indicate an involvement of Yeh fimbriae under environmental conditions, for example during survival outside their mammalian hosts. In order to confirm this hypothesis, further experiments regarding attachment, colonization and persistence on abiotic and biotic surfaces have to be done.

Further to C/U fimbriae, Curli fimbriae, and type 4 pili were investigated in this study. In contrast to previously reported contribution of Curli fimbriae to biofilm of many bacterial species (Barnhart and Chapman, 2006), surface expression of *E. coli* O157:H7 Sakai Curli fimbriae did not contribute to biofilm formation. Previous studies revealed different results on native expression of Curli fimbriae and on the impact during biofilm formation. Cookson et al. (2002) did not observe expression of Curli fimbriae under laboratory conditions, possibly due to a point mutation in the promoter region of *csgD*. In contrast, Sharma et al. (2016) showed Curli fimbriae expression in overnight cultures grown at 26°C in salt-free media, further concluding a strain isolate-dependent Curli fimbriae production. In addition, Kay et al. (2017) revealed expression of Curli fimbriae under acidic stress. Expression of Curli fimbriae encoded by *E. coli* O157:H7 Sakai is often associated with biofilm formation (Hu et al., 2013; Kudva et al., 2017), but vary between the investigated isolate. In addition to involvement of Curli fimbriae in adhesion to spinach leaves (Saldana et al., 2011), we here show contribution of Curli fimbriae in binding to mammalian MDCK cells. Thus, Curli fimbriae are potentially the link between environmental survival, and attachment and infection of mammalian hosts. Typical for type 4 pili of *E. coli* O157:H7 Sakai is formation of bundled pili which were observed before by Xicohtencatl-Cortes et al. (2007). These authors visualized type 4 pili after growth at 37°C on mica, whereas in our study non-static liquid cultures were used, potentially inhibiting bundle formation of type 4 pili. Nevertheless, Xicohtencatl-Cortes et al. (2009) described type 4 pili-mediated invasion of various epithelial cells including HeLa cells. This is in line with our finding of type 4 pili expression being involved in adhesion to HeLa cells and partly in adhesion to MDCK cells, potentially mediated by binding to laminin and fibronectin.

**FIGURE 4:**
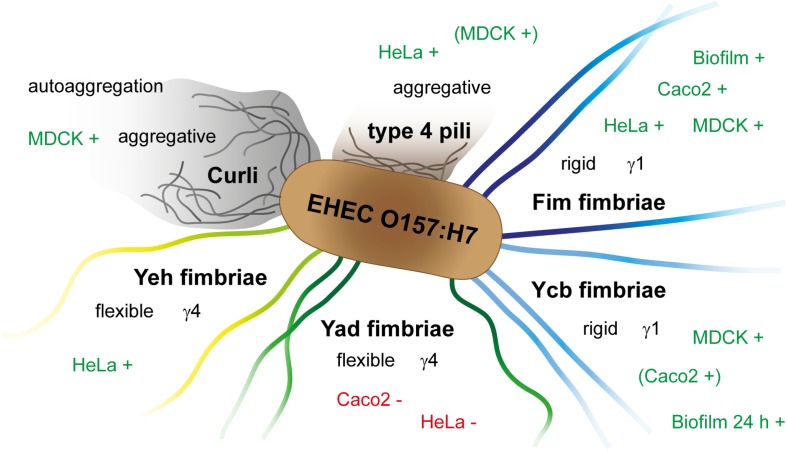
Schematic overview of contribution of *E. coli* O157:H7 Sakai fimbriae, Curli and type 4 pili to adhesion to various cell types, biofilm formation, and autoaggregation. +, increased adhesion/formation; −, decreased adhesion.

In this study, we visualized Fim, Ycb, Yad, Yeh, and Curli fimbriae and type 4 pili of *E. coli* O157:H7 Sakai, unraveled their impact in adhesion to HeLa, MDCK, and Caco2 cells, and their contribution to biofilm formation (summarized in Figure 4). Our work enables further investigation of so far uncharacterized adhesive structures of *E. coli* O157:H7 Sakai, and should lead to better understanding of pathogenicity and virulence factors of this important human pathogen.

## Materials and Methods

### Bacterial Strains and Growth Conditions

Bacterial strains used in this study are listed in Table 1. Routinely, bacteria were grown aerobically in LB (lysogeny broth) medium or on LB agar plates. If necessary for the maintenance of plasmids, carbenicillin (50 μg/ml), kanamycin (50 μg/ml), and/or chloramphenicol (12.5 μg/ml) were added to the media. For induction of the Tet-on system, overnight (o/n) cultures were diluted 1:31 in fresh LB medium in glass test tubes, anhydrotetracycline (AHT) was added to 10 ng/ml final concentration, and bacteria were subcultured for 3.5 h with aeration by continuous rotation in a roller drum at 60 rpm.

**TABLE 1:** Bacterial strains used in this study.

**Designation**	**Relevant characteristics**	**References**
ORN172	Δ*fimAICDHF*::*aph*	Woodall et al., 1993
NEBα	Cloning strain	New England Biolabs
TOP10		Invitrogen (Thermo Fisher)
SR11 Δ12	Δ*fimAICDHF*Δ*stbABCD*Δ*sthABCDE* Δ*stfACDEFG*Δ*stiABCH*Δ*bcfABCDEFGH* Δ*safABCD*Δ*pefACD*orf5orf6 Δ*stcABCD* Δ*stjEDCBA*Δ*stdAB*Δ*lpfABCDE*::KSac	Elpers et al., 2020
*S*. Typhimurium NCTC12023	*S*. Typhimurium wild type	NCTC
MvP813	Δ*invC*::*aph*	Gerlach et al., 2008

### Construction of Strains and Plasmids

Genes encoding various fimbrial operons were amplified from genomic DNA of EHEC *E. coli* O157:H7 Sakai. For design of plasmids for Tet-on expression, genome sequences were retrieved under accession numbers NC_002695 (chromosome), NC_00218 (plasmid pO157), and NC_002127 (plasmid pOSAK1). Plasmids are listed in Table 2 and were generated by Gibson assembly (GA) according to manufacturer’s instructions (NEB) using p4392 as vector by replacing the *fimAICDHF* operon by operons of interest. Oligonucleotides used are listed in Table 3. For the replacement of antibiotic resistance of pGEN-*lux* from carbenicillin to chloramphenicol the vector pGEN-*lux* was amplified lacking the carbenicillin resistance and the gene for chloramphenicol resistance was amplified from pKD3.

**TABLE 2:** Plasmids used in this study.

**Plasmid**	**Working name**	**Relevant characteristics, resistance**	**NC_002695 designation**	**References**
pGEN-*lux*	–	P_EM__7_::*luxCDABE* Carb^R^	–	Lane et al., 2007
pKD3	*–*	*cat cassette* FRT Carb^R^ Cm^R^	–	Datsenko and Wanner, 2000
p4392	Fim-STM	pWSK29 *tetR* P*_*tetA*_*::*fimAICDHF* of *S*. Typhimurium Carb^R^	–	Hansmeier et al., 2017
p4545	Type 4 pili	*tetR* P*_*tetA*_*::ECs0110-ECs0112 (type 4 pili) Carb^R^	ECs0110-ECs0112	This study
p4547	Fim	*tetR* P*_*tetA*_*::ECs5273-ECs5279 (Fim fimbriae) Carb^R^	ECs5273-ECs5279	This study
p4629	Yad	*tetR* P*_*tetA*_*::ECs0139-ECs0145 (Yad fimbriae) Carb^R^	ECs0139-ECs0145	Schulte et al., 2019
p4916	Ycb	*tetR* P*_*tetA*_*::ECs1021-ECs1028 (Ycb fimbriae) Carb^R^	ECs1021-ECs1028	This study
p4922	*–*	*tetR P_*tetA*_*::ECs1419-ECs1420-*csgC* Carb^R^	ECs1419-ECs1420	This study
p4923	Curli	*tetR* P*_*tetA*_*::ECs1414-ECs1419 (Curli fimbriae) Carb^R^	ECs1414-ECs1419	This study
p5317	Yeh	*tetR* P*_*tetA*_*::ECs2914-ECs2918 (Yeh fimbriae) Carb^R^	ECs2914-ECs2918	This study
p5236	Lux	P_EM__7_::*luxCDABE*-P*_*cat*_*::*cat* Cm^R^	–	This study

**TABLE 3:** Oligonucleotides used in this study.

**Designation**	**Sequence (3′->5′)**	**Purpose**
Vf-pWSK29	GAATTCCTGCAGCCCGGGG	Vector p4392 and p4922
Vr-pWSK29-Ptet-rev	TTCACTTTTCTCTATCACTGATAGGGAGTGGTAAAATAACTCT	Vector p4392
STEC-GA-pWSK29-hcp-rev	ACTAGTGGATCCCCCGGGCTGCAGGAATTCTTATCCCATCCCACTCATCG	Type 4 pili GA
STEC-Hcp-GA-Ptet-for	CACTCCCTATCAGTGATAGAGAAAAGTGAAATGGACAAGCAACGCGGTTT	Type 4 pili GA
Sakai-GA-pWSK29-fim-rev	ACTAGTGGATCCCCCGGGCTGCAGGAATTCTTATTGATAAACAAAAGTCACGCCAATAAT	Fim fimbriae GA
Sakai-Fim-GA-Ptet-for	CACTCCCTATCAGTGATAGAGAAAAGTGAAATGAAAATTAAAACTCTGGCAATCGT	Fim fimbriae GA
Vf-Sakai-Ycb-operon	CACTCCCTATCAGTGATAGAGAAAAGTGAAATGATAACGATGAAAAAAAGTGTAT	Ycb fimbriae GA
Vr-Sakai-Ycb-operon	CCCCGGGCTGCAGGAATTCTCAGGTTAATGTAATAAGGCG	Ycb fimbriae GA
Vf-C227-csgBAC	CACTCCCTATCAGTGATAGAGAAAAGTGAAATGAAAAACAAATTGTTATTTATGATG	Curli fimbriae GA
Vr-C227-csgEFG	CCCCGGGCTGCAGGAATTCTCAGGATTCCGGTGGAAC	Curli fimbriae GA
Vr-C227-csgBAC	CCCCGGGCTGCAGGAATTCTTAAGACTTTTCTGAAGAGGGC	Curli fimbriae GA
Vr-assembly-C227-csgBAC-csgEFG	TTAAGACTTTTCTGAAGAGGGCGGCCATTGT	Vector p4922
Vf-C227-csgEFG	CAATGGCCGCCCTCTTCAGAAAAGTCTTAAAGCCATGAAACGTTATTTACG	Curli fimbriae GA
1r-Sakai_Ptet-2913-9218-fimbriae-pWSK29	CCGGGCTGCAGGAATTCTTAATCGTATTTCACGTTGATTAATG	Yeh fimbriae GA
1f-Sakai_Ptet-Yeh-fimbriae-pWSK29	CCCTATCAGTGATAGAGAAAAGTGAAATGAATAAGTATTGGTTGTCAG	Yeh fimbriae GA
Vf-pFPV25.1	AATCTCCTGCAGGCATGCAA	Vector pGEN-*lux*
Vr-pFPV25.1	ATGTATATCTCCTTCTTAAATCTAGAGGATCT	Vector pGEN-*lux*
1r-pKD3	GACGAAAGGGCCTCGTGACCTACCTGTGACGGA	*cat* GA
1f-pKD3	ATATGAGTAAACTTGGTCTGACAGTCA TCGCAGTACTGTTGT	*cat* GA

### Transmission Electron Microscopy

For the visualization of fimbriae by transmission electron microscopy (TEM), subcultured bacteria were pelleted at 1,000 × g for 5 min and supernatant was removed immediately. Bacteria were washed once with ultrapure water (MilliQ water). Pelleted bacteria were then resuspended in 3% PFA/PBS and fixed for 20 min at RT. Bacteria were pelleted again and resuspended in MilliQ water. 5 μl of bacterial suspension were dropped on 100 mesh formvar/carbon grids which were glow discharged immediately before use by an easiGlow instrument (Pelco) for 10 sec with 15 mA. Bacteria were further stained with 1% PTA pH 7.4 for 2 min. Grids were washed thrice in MilliQ water and further processed for TEM (Zeiss 902, 80 keV).

### Cell Culture

HeLa cells (obtained from Cell Lines Service CLS, Heidelberg), Caco2 cells (C2BBE1, American Type Culture Collection, ATCC) and MDCK cells (subline pf, obtained from Prof. Dr. M. Goppelt-Struebe, Med. Klinik 4, Universitätsklinikum Erlangen) were cultured as described before (Gerlach et al., 2008). For luminescence-mediated adhesion assays, and lysate-mediated adhesion and invasion assays, cells were seeded in white 96-wells plates (F96 MicroWell^TM^ Plates, PS Nunclon^TM^D, white; unless otherwise stated) at a density of 1.7 × 10^4^ cells per well for HeLa cells, and 1.6 × 10^4^ cells per well for Caco2 and MDCK cells. HeLa cells were cultured for 24 h, MDCK cells were cultured for 1 week, whereas Caco2 cells were cultured for 3 weeks to ensure polarization of cells. The media for MDCK and Caco2 cells were supplemented with penicillin and streptomycin. The medium was changed to antibiotic-free medium at least 3 h before infection.

### Autoaggregation

Autoaggregation of bacteria after expression of various fimbriae was analyzed by microscopic inspection. A 3.5 h subculture was diluted to ca. 3 × 10^8^ bacteria/ml in PBS and 7 μl bacterial suspension were imaged by an Axio Observer with brightfield microscopy with a 40× objective (Zeiss). Images were recorded with an AxioCam and processed with ZEN 2012.

### Luminescence-Mediated Adhesion Assay

For the luminescence mediated adhesion assay, white 96-well plates (F96 MicroWell^TM^ Plates, PS Nunclon^TM^D, white) were used to enable selective detection of each well. Caco2, HeLa, and MDCK cells were infected with 3.5 h subcultures of *E. coli* with a multiplicity of infection (MOI) of 25. Infection was synchronized by centrifugation for 5 min at 500 × g, and luminescence was measured using a CHAMELEON V (Hidex) microplate reader. Further infection took place for 55 min at 37°C in an atmosphere of 5% CO_2_. After infection, cells were washed three times with prewarmed PBS in case of HeLa and MDCK cells, or with prewarmed Caco2 medium. Luminescence measurements took place in finally 200 μl PBS or medium. Adhesion rates were determined by the quotient of starting luminescence and final luminescence. Furthermore, adhesion rates were normalized to 100% of background strain ORN172 [Lux].

### Adhesion and Invasion Assay by CFU Determination

CaCo2, HeLa, and MDCK cells were infected with 3.5 h subcultures of *E. coli* with a multiplicity of infection (MOI) of 25. Infection was synchronized by centrifugation at 500 × g for 5 min, and infected cells were incubated for 25 min at 37°C in an atmosphere of 5% CO_2_. The cells were washed three times with prewarmed PBS in case of HeLa and MDCK cells, or with prewarmed Caco2 medium, and 500 μl medium containing 100 μg/ml gentamicin was applied to each well. After incubation for 1 h, medium was removed and cells were washed three times with prewarmed PBS for HeLa and MDCK cells, or with prewarmed Caco2 medium. Cells were lysed for 10 min by addition of 0.1% deoxycholate in PBS, or 0.1% Triton X-100 in PBS for MDCK and Caco2 cells, respectively. Serial dilution of inoculum and lysates were plated on MH plates to determine CFU. Percentages of invaded bacteria were calculated. For adhesion assay infection took place after centrifugation for 1 h at 37°C in an atmosphere of 5% CO_2_. The cells were washed three times with prewarmed PBS in case of HeLa and MDCK cells or with prewarmed Caco2 medium and lysed by 0.1% deoxycholate/PBS for MDCK and Caco2 cells or by 0.1% Triton X-100 in PBS for 10 min. Serial dilution of inoculum and lysates were plated on MH plates to determine CFU. Percentages of adhered bacteria were calculated.

### Crystal Violet Assay

For the quantification of biofilm formation, bacterial strains were cultivated as described before. The bacterial cultures were diluted to 3 × 10^8^ bacteria/ml in LB media (with antibiotics if necessary) and 200 μl bacterial solution were filled per well in a 96-well plate (Polystyrol, flat bottom without coating). Each sample was measured for OD_595_ in triplicates using a Plate CHAMELEON V. The 96-well plates were incubated in dark in a humid chamber at 30°C or 37°C for 24 h to 72 h. After incubation, OD_595_ was measured to confirm an equal growth of bacteria. Wells were washed three times with PBS and air dried for at least 2 h at RT. Biofilm was stained with 0.1% crystal violet in H_2_O_dd_ for 5 min at RT on a rocking plate. Wells were washed again three times with H_2_O_dd_ and the bound crystal violet was resolved by adding 200 μl 100% EtOH for 20 min at RT on a rocking plate. Finally, the OD_595_ was measured.

### Flow Cytometry

For analysis of surface expression of Fim fimbriae of STM by detection of FimA by flow cytometry, ca. 6 × 10^8^ bacteria were washed in PBS and then fixed with 3% paraformaldehyde in PBS for 20 min. Bacteria were incubated in blocking solution (2% goat serum in PBS) for 30 min, followed by staining with rabbit α-FimA antiserum, diluted 1:1,000 in blocking solution for 2 h, and detection by goat α-rabbit IgG antibody coupled to Alexa-Fluor488, diluted 1:2,000 in blocking solution for 1 h. Bacteria were analyzed using an Attune NxT Flow Cytometer (Thermo Fisher) and Attune NxT Software version 2.7. A strain lacking the respective adhesive structure was used as a negative control for gating.

### Statistical Analyses

Statistical significances were calculated by Student’s t-test (two-sided) and performed with Excel (Microsoft Office 2016). Significances are indicated as follows: n.s., not significant; ^∗^*p* < 0.05; ^∗∗^*p* < 0.01; ^∗∗∗^*p* < 0.001. At least three biological replicates were included with each three technical replicates.

## Data Availability Statement

The datasets generated for this study are available on request to the corresponding author.

## Author Contributions

LE and MH designed the research, analyzed the data, and wrote the manuscript. LE performed the research.

## Conflict of Interest

The authors declare that the research was conducted in the absence of any commercial or financial relationships that could be construed as a potential conflict of interest.

## Supplementary Material

FIGURE S1 Ultrastructure of *Escherichia coli* O157:H7 Sakai fimbriae after surface expression *in *E. coli** ORN172. Shown are only non-induced bacteria corresponding to Figure 1. Each panel contains an overview of bacteria and two hatched boxes indicate enlarged depicted area (i and ii). Scale bars, 250 nm and 100 nm in overviews and details, respectively.

FIGURE S2 Establishment of a luminescence-based adhesion assay. **(A)** Measurement of relative bioluminescence as a function of amounts of bacteria of TOP10 [pGEN-*lux*] or NEBα [Lux] diluted in PBS. **(B,C)** Comparison of adhesion rates of SR11 Δ12 [Lux] and SR11 Δ12 [Lux] [Fim-STM] to MDCK cells determined by lysis and plating or luminescence measurements. Further normal transparent cell culture plates (NP) were compared to white cell culture plates (WP). The percentages of adherent bacteria of inoculum are shown in **(B)**, and adhesion levels were normalized to 100% of SR11 Δ12 [Lux] for each condition **(C)**. MDCK cells were infected at a MOI of 25 for 55 min at 37°C in an atmosphere containing 5% CO_2_. Infection was synchronized by centrifugation of 5 min at 500 × *g*. Cells were washed three times and either lysed with 0.1% deoxycholate/PBS and plated onto agar plates or luminescence were measured using a microplate reader. **(D)** To determine the optimal number of washing steps for measurement of adhered luminescent bacteria to cells, luminescence was measured after each washing step. For this, MDCK cells were infected at MOI of 25 under the conditions described before. Adhesion levels were normalized to 100% of relative luminescence for each strain at infection time point 0 h. The phenotype of STM Fim was clear visible after three washing steps in comparison to background strain SR11 Δ12 [Lux] and non-induced SR11 Δ12 [Lux] [Fim-STM]. **(E,F)** *E. coli* strain ORN172 [Lux] and ORN172 [Lux] [Fim-STM] were checked by lysis for possible invasion in HeLa and Caco2 cells under the conditions used for the adhesion assay. Further STM WT and STM Δ*invC* deletion mutant were used as controls. Infection temperature of 37°C was compared to room temperature (RT) and 4°C, adhesion levels were compared to invasion levels which were determined by a Gentamicin protection assay as described before (Gerlach et al., 2007). Shown are the adhesion and invasion levels in % of inoculum. For HeLa and Caco2 cells, no invasion of ORN172 strains were observed. Phenotype of STM Fim adhering to HeLa and Caco2 cells were most distinct at infection temperature of 37°C.

FIGURE S3 Quantification of Fim fimbriae of STM expression in ORN172 [Lux] [Fim-STM]. Tet-on expression of Fim fimbriae of STM was measured by flow cytometry analysis. Fimbriae were detected by antibody using rabbit α-FimA (1:1,000) and goat α-rabbit-Alexa488 (1:2,000). Overlays of the measured fluorescence intensities are shown in **(A)**, the percentages of Alexa488-positive bacteria are given in **(B)**.

FIGURE S4 Effect of *E. coli* O157:H7 Sakai Curli expression on biofilm formation. *E. coli* ORN172 [Lux] was used as host strain harboring the vector (none, black bars), or plasmids for Tet-on expression of STM Fim fimbriae (Fim-STM, light gray bars), or *E. coli* O157:H7 Sakai Curli (Curli, dark gray bars). Biofilm assays were performed as described for Figure 3, but assays were performed at 30°C with incubation of 24 h **(A)** or 72 h **(B)**.

FIGURE S5 Micrographs of ORN172 [Lux] with AHT-induced expression of *E. coli* O157:H7 Sakai fimbriae. Tet-on expression was induced by addition of 10 ng/ml AHT, or not induced. After subculture for 3.5 h, cultures were diluted to 1 × 10^8^ bacteria/ml in PBS. Bacteria were visualized by bright-field microscopy using an Axio Observer system with a 40× objective (Zeiss). Images were recorded with an AxioCam and data were processed with ZEN 2012. Scale bars; 20 μm.
