# Supplementary figures and images for: Expression and Functional Characterization of Various Chaperon-Usher Fimbriae, Curli Fimbriae, and Type 4 Pili of Enterohemorrhagic Escherichia coli O157:H7 Sakai
Source: Front Microbiol. 2020 Mar 20;11:378. doi: 10.3389/fmicb.2020.00378 (PMC7098969; doi:10.3389/fmicb.2020.00378)

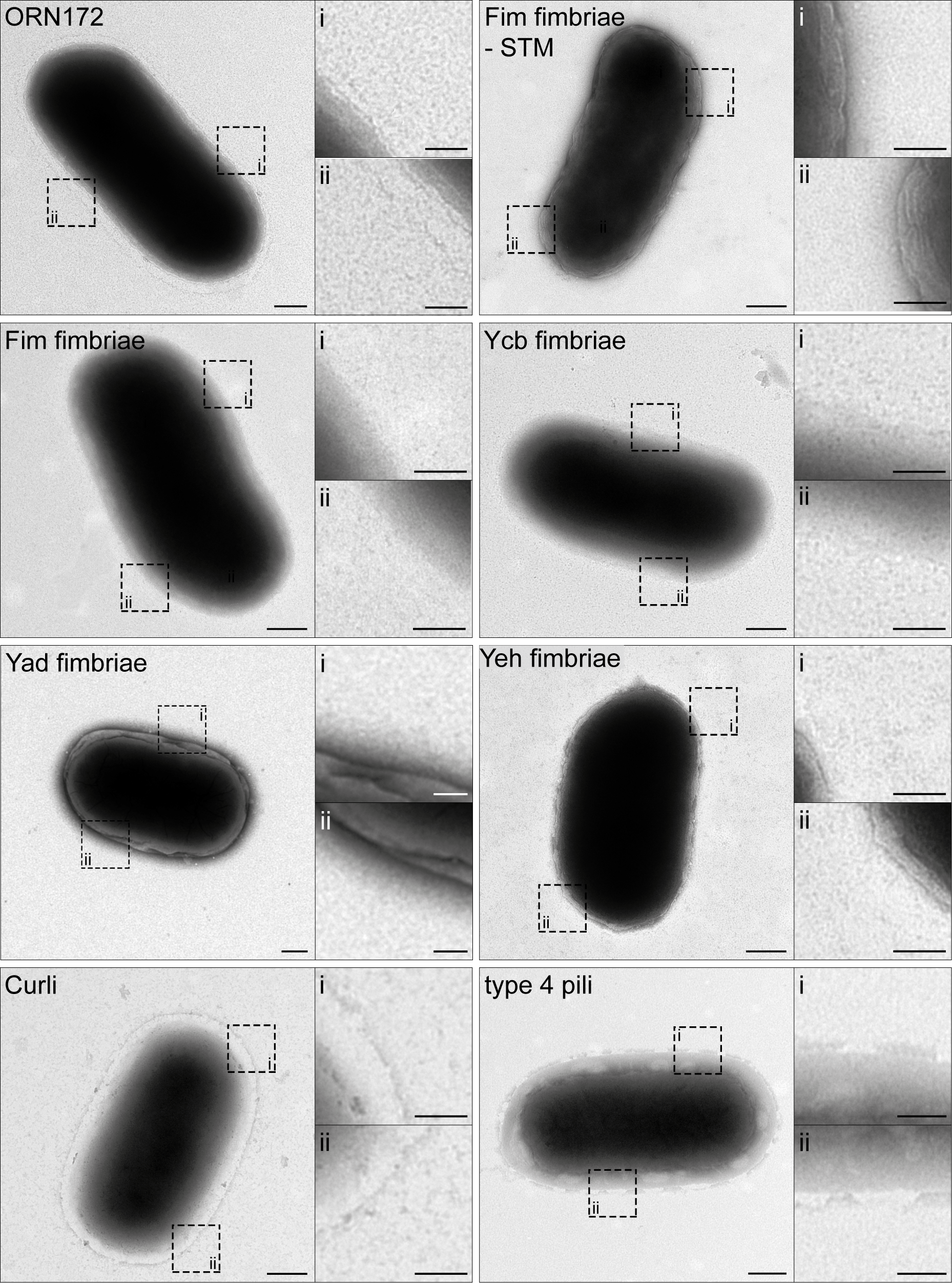

Supplement: FIGURE S1 — Ultrastructure of Escherichia coli O157:H7 Sakai fimbriae after surface expression in E. coli ORN172. Shown are only non-induced bacteria corresponding to Figure 1. Each panel contains an overview of bacteria and two hatched boxes indicate enlarged depicted area (i and ii). Scale bars, 250 nm and 100 nm in overviews and details, respectively. [file Image_1.TIF]

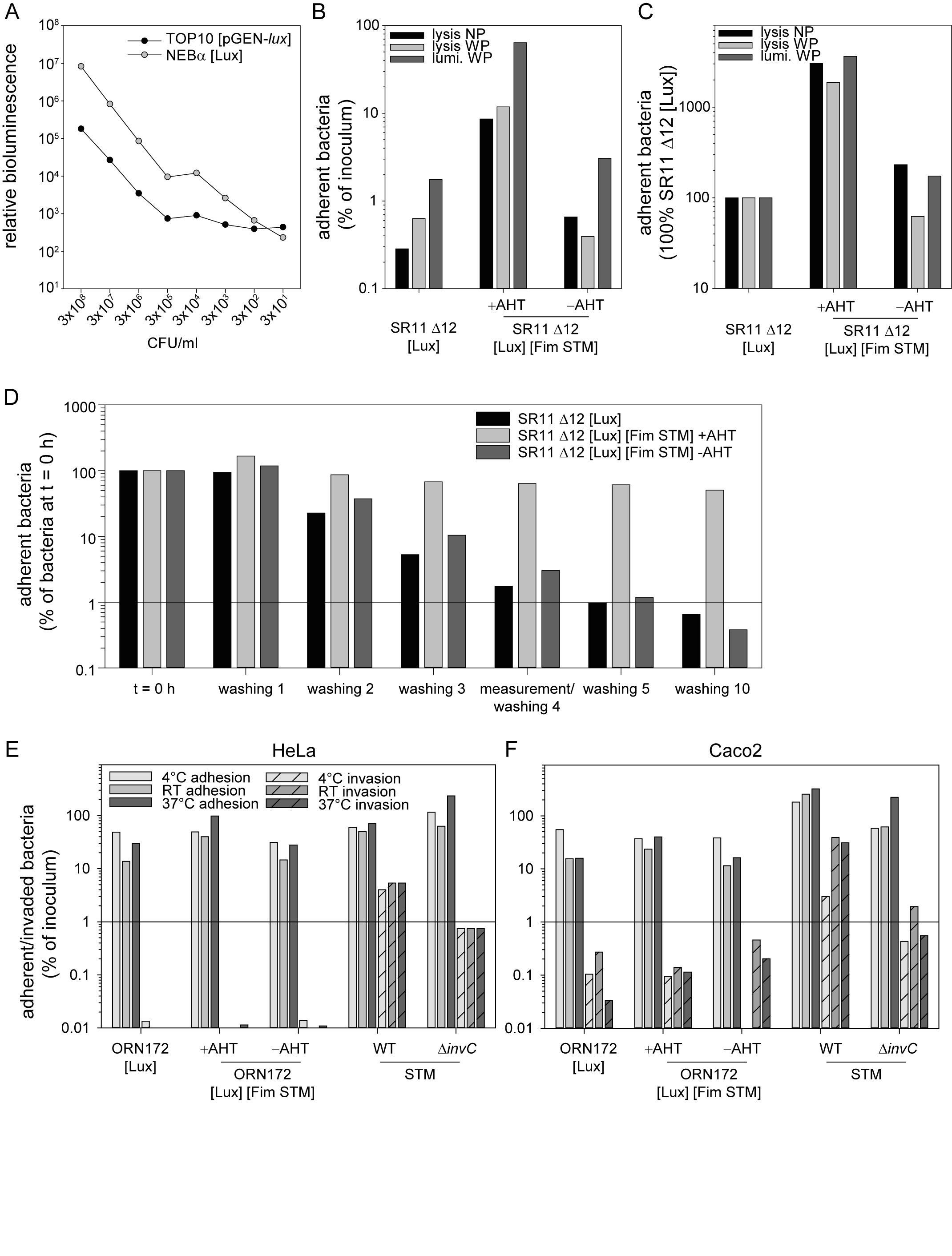

Supplement: FIGURE S2 — Establishment of a luminescence-based adhesion assay. (A) Measurement of relative bioluminescence as a function of amounts of bacteria of TOP10 [pGEN-lux] or NEBα [Lux] diluted in PBS. (B,C) Comparison of adhesion rates of SR11 Δ12 [Lux] and SR11 Δ12 [Lux] [Fim-STM] to MDCK cells determined by lysis and plating or luminescence measurements. Further normal transparent cell culture plates (NP) were compared to white cell culture plates (WP). The percentages of adherent bacteria of inoculum are shown in (B), and adhesion levels were normalized to 100% of SR11 Δ12 [Lux] for each condition (C). MDCK cells were infected at a MOI of 25 for 55 min at 37°C in an atmosphere containing 5% CO2. Infection was synchronized by centrifugation of 5 min at 500 × g. Cells were washed three times and either lysed with 0.1% deoxycholate/PBS and plated onto agar plates or luminescence were measured using a microplate reader. (D) To determine the optimal number of washing steps for measurement of adhered luminescent bacteria to cells, luminescence was measured after each washing step. For this, MDCK cells were infected at MOI of 25 under the conditions described before. Adhesion levels were normalized to 100% of relative luminescence for each strain at infection time point 0 h. The phenotype of STM Fim was clear visible after three washing steps in comparison to background strain SR11 Δ12 [Lux] and non-induced SR11 Δ12 [Lux] [Fim-STM]. (E,F) E. coli strain ORN172 [Lux] and ORN172 [Lux] [Fim-STM] were checked by lysis for possible invasion in HeLa and Caco2 cells under the conditions used for the adhesion assay. Further STM WT and STM ΔinvC deletion mutant were used as controls. Infection temperature of 37°C was compared to room temperature (RT) and 4°C, adhesion levels were compared to invasion levels which were determined by a Gentamicin protection assay as described before (Gerlach et al., 2007). Shown are the adhesion and invasion levels in % of inoculum. For HeLa and Caco2 cells, no i [file Image_2.TIF]

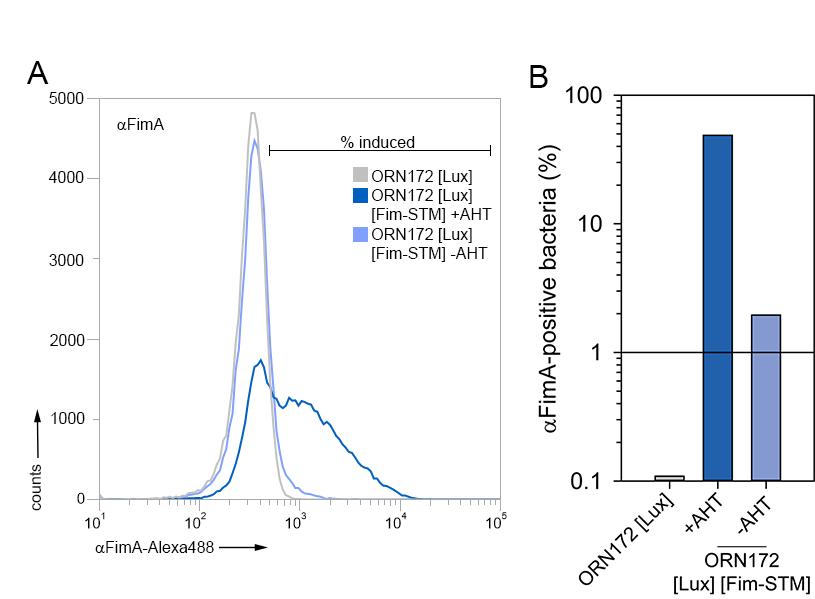

Supplement: FIGURE S3 — Quantification of Fim fimbriae of STM expression in ORN172 [Lux] [Fim-STM]. Tet-on expression of Fim fimbriae of STM was measured by flow cytometry analysis. Fimbriae were detected by antibody using rabbit α-FimA (1:1,000) and goat α-rabbit-Alexa488 (1:2,000). Overlays of the measured fluorescence intensities are shown in (A), the percentages of Alexa488-positive bacteria are given in (B). [file Image_3.TIF]

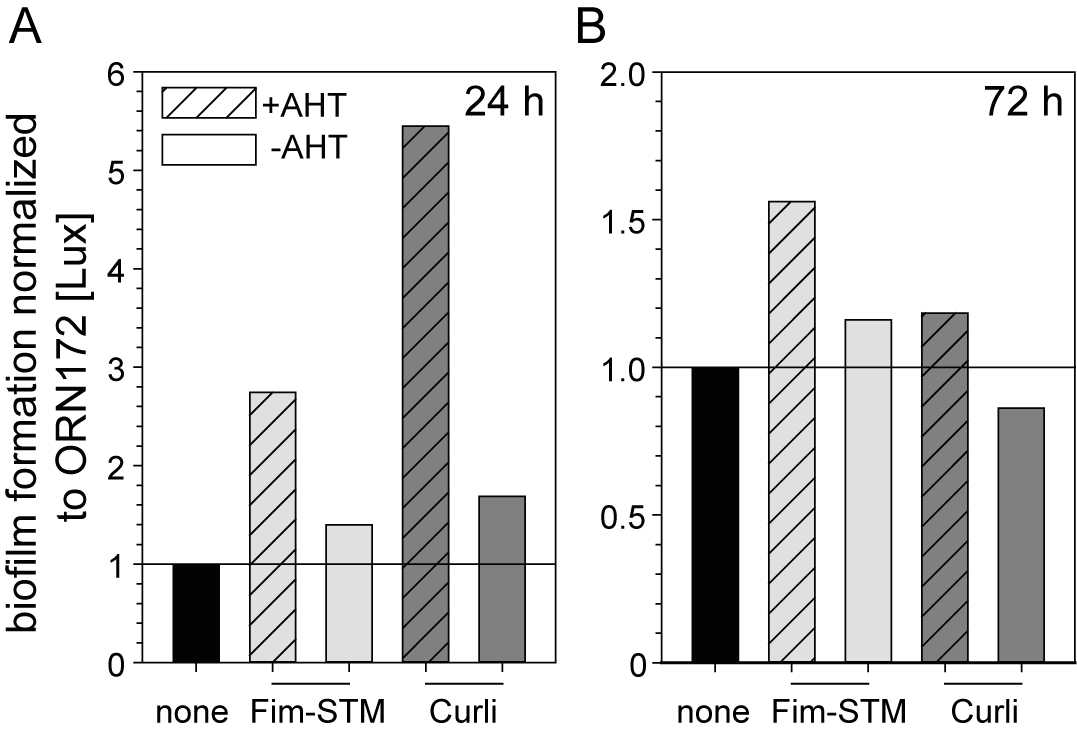

Supplement: FIGURE S4 — Effect of E. coli O157:H7 Sakai Curli expression on biofilm formation. E. coli ORN172 [Lux] was used as host strain harboring the vector (none, black bars), or plasmids for Tet-on expression of STM Fim fimbriae (Fim-STM, light gray bars), or E. coli O157:H7 Sakai Curli (Curli, dark gray bars). Biofilm assays were performed as described for Figure 3, but assays were performed at 30°C with incubation of 24 h (A) or 72 h (B). [file Image_4.TIF]

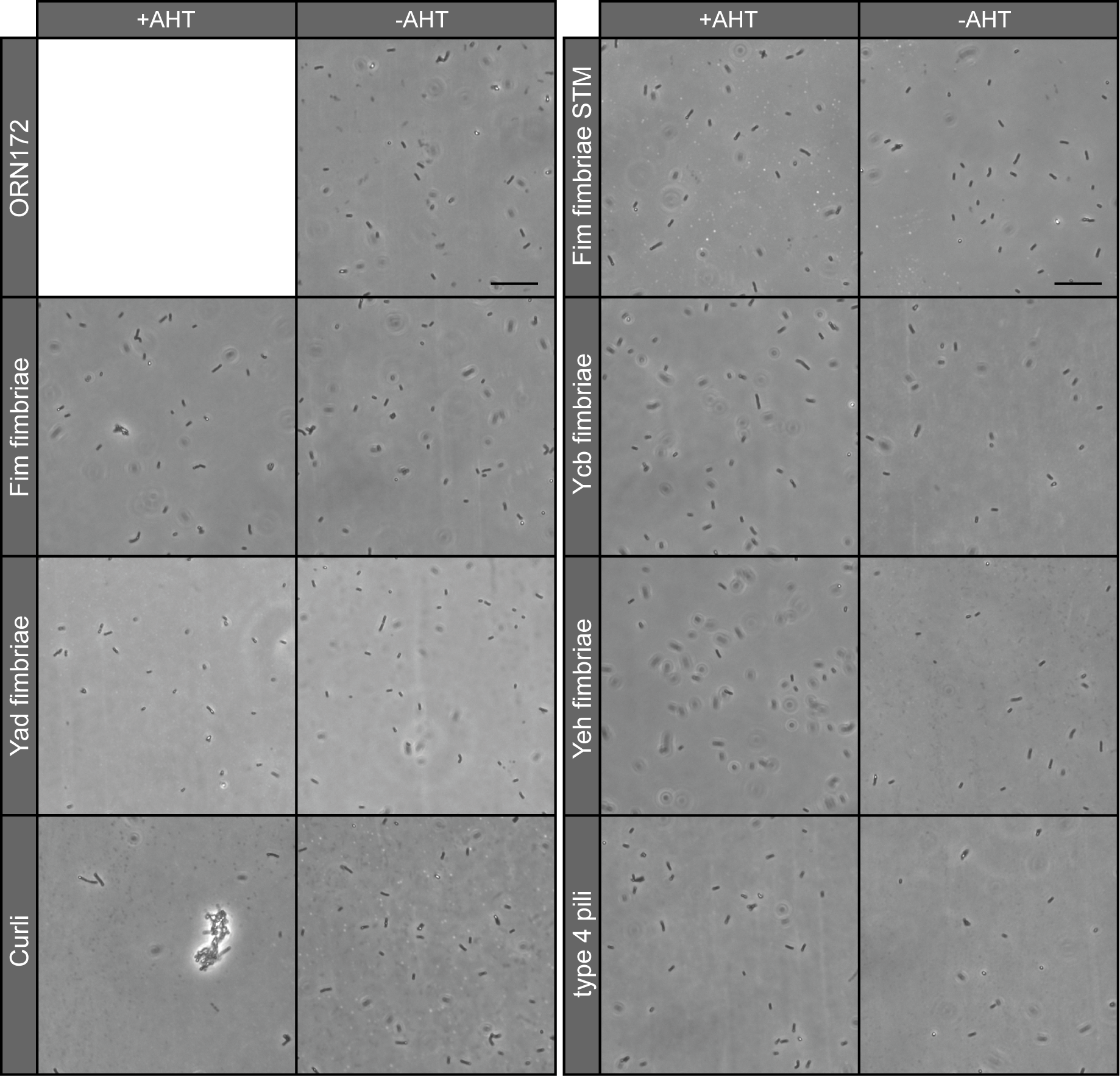

Supplement: FIGURE S5 — Micrographs of ORN172 [Lux] with AHT-induced expression of E. coli O157:H7 Sakai fimbriae. Tet-on expression was induced by addition of 10 ng/ml AHT, or not induced. After subculture for 3.5 h, cultures were diluted to 1 × 10^8^ bacteria/ml in PBS. Bacteria were visualized by bright-field microscopy using an Axio Observer system with a 40× objective (Zeiss). Images were recorded with an AxioCam and data were processed with ZEN 2012. Scale bars; 20 μm. [file Image_5.TIF]
